# Improvement of Premium Oil Soybean Variety Heinong 551 with Integrating Conventional Hybridization and Gamma Radiation

**DOI:** 10.3390/life15101616

**Published:** 2025-10-16

**Authors:** Xiulin Liu, Xueyang Wang, Kezhen Zhao, Chunlei Zhang, Fengyi Zhang, Rongqiang Yuan, Sobhi F. Lamlom, Honglei Ren, Bixian Zhang

**Affiliations:** 1 Soybean Research Institute of Heilongjiang Academy of Agriculture Sciences, Harbin 150086, China; liuxiulin1002@126.com (X.L.); hljsnkywxy@163.com (X.W.); zhaokz928@163.com (K.Z.); zhangchunlei1989@yeah.net (C.Z.); ddszhangfy2019@163.com (F.Z.); yrq18846121189@163.com (R.Y.); sobhifaid@alexu.edu.eg (S.F.L.); renhonglei2022@163.com (H.R.); 2 Plant Production Department, Faculty of Agriculture Saba Basha, Alexandria University, Alexandria 21531, Egypt; 3 Institute of Biotechnology of Heilongjiang Academy of Agricultural Sciences, Harbin 150023, China

**Keywords:** *Glycine max*, high-oil variety, multi-location evaluation, mutation breeding, soybean breeding, yield stability

## Abstract

Meeting the growing demand for vegetable oil while promoting agricultural sustainability in Northeast China requires developing high-yield, high-oil-content soybean varieties. We present the comprehensive development and evaluation of Heinong 551, an innovative soybean variety created through an integrated approach of conventional breeding methods and radiation-induced mutation techniques. The breeding program began with hybridization between Heinong 44 (the maternal parent) and Hefeng 47 (the paternal parent), followed by targeted exposure to ^60^Co gamma radiation at 130 Gy to induce beneficial mutations. Using systematic selection protocols over five generations from 2012 to 2016, we identified superior lines that underwent rigorous multi-location testing across seven sites in Heilongjiang Province during 2020–2021. Field evaluation results showed consistent performance, with Heinong 551 achieving average yields of 2901 kg/ha and 3142 kg/ha in those years, representing significant gains of 10. 6% and 11.0. 0% compared to standard control varieties. The cultivar maintained stable phenological traits with a reliable 120-day maturation period and demonstrated strong environmental adaptability across different growing conditions. Biochemical analysis revealed excellent nutritional value, with 39.45% crude protein and 21.69% crude fat, reaching a combined protein–fat percentage of 61.14%. Quality tests confirmed superior seed integrity, with sound seed rates over 97% and minimal pest or disease damage. Disease resistance assessments showed moderate tolerance to gray leaf spot while maintaining excellent overall plant health, with no signs of viral infections or nematode infestations during testing. Heinong 551 has received official approval for cultivation in Heilongjiang Province’ s second accumulated temperature zone, characterized by thermal units ≥2550 °C above a 10 °C threshold. This represents significant progress in high-oil soybean variety development, illustrating the success of combining traditional breeding methods with modern mutation technology.

## 1. Introduction

Soybean [*Glycine max* (L.) Merr.] represents one of humanity’s most critical agricultural commodities, serving simultaneously as a primary source of plant protein and vegetable oil while supporting diverse industrial applications from biodiesel production to pharmaceutical compounds [1,2]. Global soybean production has experienced unprecedented growth, reaching 395.1 million metric tons in the 2023–2024 season and accounting for over 60% of worldwide oilseed output [3]. This expansion reflects not merely increased acreage but fundamental shifts in dietary patterns, with growing populations in developing nations driving protein demand while simultaneously increasing requirements for vegetable oils in food processing and renewable energy sectors [4,5,6].

The fundamental challenge in soybean improvement centers on the well-documented negative correlation between oil and protein content, a relationship that has constrained breeding progress for decades [7,8,9]. This biochemical antagonism, with correlation coefficients typically ranging from −0.6 to −0.8, reflects complex metabolic competition between lipid biosynthesis and protein accumulation pathways during seed development [10]. Traditional breeding approaches have typically achieved gains in one trait at the expense of the other, limiting the potential for simultaneous improvement despite growing market demands for varieties combining high oil content with adequate protein levels [11,12,13]. Recent advances in plant physiology have revealed that this trade-off stems from shared carbon and energy resources during seed filling, with regulatory mechanisms controlling the allocation between oil and protein synthesis [14,15]. Transcriptomic studies have identified key metabolic control points where lipid and protein biosynthetic pathways compete for common precursors, particularly during the critical period of rapid seed filling. Understanding these physiological constraints provides opportunities for strategic intervention through breeding approaches that can potentially circumvent or minimize trade-off effects [11,16,17]. The economic implications of overcoming this trade-off are substantial. Each percentage point increase in oil content can improve processing efficiency by 3–5% while reducing extraction costs, directly impacting profitability across the value chain. Simultaneously, maintaining protein levels above 38% ensures continued utility for livestock feed applications, preserving market access and price premiums. Varieties successfully combine high oil content (>21%) with competitive protein levels (>38%) represent significant commercial breakthroughs, with potential market advantages exceeding 15–20% over conventional varieties.

The biochemical antagonism between oil and protein accumulation in soybean seeds reflects fundamental competition for carbon and energy resources during seed development [18,19]. During the rapid seed-filling phase (R5–R7 growth stages), photosynthate allocation is partitioned between lipid biosynthesis pathways, primarily occurring in oil bodies, and protein synthesis occurring in protein storage vacuoles [20]. Transcriptomic analyses have identified critical regulatory nodes where these pathways compete, including acetyl-CoA carboxylase (ACCase) for lipid synthesis and asparagine synthetase for protein accumulation [21,22]. Quantitative trait locus (QTL) mapping studies have identified multiple genomic regions controlling seed oil content on chromosomes 5, 9, 15, and 20, with several QTLs showing pleiotropic effects on both oil and protein content [23,24]. Major effect QTLs such as *cqSeed oil-001* on chromosome 20 explain up to 15% of phenotypic variance for oil content, while simultaneously affecting protein content in the opposite direction [25]. Candidate genes within these QTL intervals include fatty acid desaturases, diacylglycerol acyltransferases (DGAT), and transcription factors regulating lipid metabolism [26]. Recent genome-wide association studies (GWAS) using diverse soybean germplasm have revealed that natural variation in seed composition traits is controlled by numerous small-effect loci, making simultaneous improvement through conventional breeding particularly challenging [27]. This genetic architecture necessitates innovative approaches to generate novel allelic variants not present in existing breeding populations, positioning mutagenesis as a complementary strategy to conventional recombination breeding [27].

Mutation breeding, utilizing ionizing radiation to induce beneficial genetic variations, has contributed over 3400 officially registered crop varieties worldwide [28]. Yet, its application in soybean improvement remains underexplored relative to other major crops [29,30]. The technique’s success in cereals, where it has contributed to varieties accounting for millions of hectares globally, demonstrates its potential for generating novel genetic diversity that is unavailable through conventional crossing approaches. In soybeans, gamma radiation has shown particular promise for modifying seed composition traits, with optimal doses typically ranging from 100–200 Gy depending on genotype and seed moisture content [31]. The physiological basis for the effectiveness of mutation breeding lies in its ability to generate novel allelic variants that affect quantitative traits without the limitations imposed by existing genetic diversity within breeding populations. Unlike conventional breeding, which relies on recombining existing alleles, mutagenesis can create entirely new genetic variants affecting metabolic pathways, regulatory mechanisms, and developmental processes. This capability proves particularly valuable for traits exhibiting strong genetic correlations, where conventional approaches may be insufficient to break established linkages between characteristics.

Recent molecular studies have revealed that gamma radiation preferentially induces single nucleotide polymorphisms and small insertions/deletions rather than large chromosomal rearrangements, making it particularly suitable for fine-tuning metabolic pathways without disrupting essential gene functions [30,32,33]. The stochastic nature of mutation induction means that beneficial variants occur alongside neutral and deleterious mutations, necessitating systematic selection protocols to identify and stabilize desirable genetic changes while eliminating harmful effects [30].

Within China’s agricultural framework, soybean improvement carries strategic significance extending beyond typical commodity considerations to encompass national food security and economic independence objectives [34,35]. China currently imports over 100 million metric tons of soybeans annually, representing approximately 85% of domestic consumption and creating substantial dependency on international markets [29]. Heilongjiang Province emerges as China’s most critical region for domestic soybean production, contributing approximately 40% of national output while maintaining the highest average yields and oil content levels nationwide. The province’s environmental advantages include optimal thermal regimes with accumulated temperatures between 2400–2800 °C above 10 °C base, well-distributed precipitation patterns averaging 500–700 mm during growing seasons, and nutrient-rich black soils with organic matter content exceeding 3%. These conditions naturally favor oil accumulation in soybean seeds, making the region ideal for developing and testing high-oil varieties [36,37].The development of superior soybean varieties within China addresses multiple policy objectives simultaneously. Enhanced domestic production capacity reduces import dependency while supporting rural economic development in key agricultural regions. High-oil varieties specifically target the growing domestic vegetable oil market, currently supplied primarily through imports of soybeans and palm oil. Success in developing competitive high-oil, high-yielding varieties could potentially reduce import requirements by 10–15% while generating substantial economic benefits for domestic producers.

This research addresses the critical challenge of developing soybean varieties that overcome traditional oil-protein trade-offs through integration of conventional hybridization with gamma radiation mutagenesis. Our specific objectives encompass: (1) developing a novel high-oil soybean variety combining superior oil content (>21%) with competitive yield performance through integrated breeding approaches; (2) characterizing the genetic stability and environmental adaptation of mutation-derived improvements across diverse growing conditions; (3) establishing a replicable methodology for combining conventional and mutation breeding techniques in complex trait improvement programs; and (4) evaluating the commercial viability and deployment potential of the developed variety within China’s soybean production systems.

## 2. Materials and Methods

### 2.1. Plant Materials and Breeding Strategy

The breeding program for Heinong 551 utilized two high-oil soybean varieties as parental lines, selected based on their superior oil content, agronomic performance, and complementary characteristics. The maternal parent, Heinong 44, was previously developed through hybridization of Ha85-6437 × Jilin 20 and exhibited exceptional quality characteristics with 23.01% fat content, 36.06% protein content, and yield performance of 2936.6 kg/ha representing a 13.9% increase over control variety Hefeng 25. The paternal parent, Hefeng 47, was developed through radiation treatment of F_2_ generation material from He9229 derived from Hefeng 35 × Gong84112-1-3 followed by consecutive generation selection, containing 22.85% fat and 38.11% protein with yield performance of 2560.8 kg/ha representing a 13.1% increase over control variety Hefeng 35.

The selection of both high-oil parents, rather than parents from opposite ends of the oil content distribution, was based on the breeding objective of maximizing oil content while maintaining protein quality. Previous research has demonstrated that crossing two high-oil parents increases the probability of recovering transgressive segregants with oil content exceeding both parents when combined with mutagenesis techniques. This strategy also ensured that both parents contributed favorable alleles for oil biosynthesis pathways, creating a genetic foundation for enhanced oil accumulation in subsequent generations while maintaining the necessary genetic diversity for effective selection.

### 2.2. Hybridization and Mutation Breeding Protocol

**Figure 1:**
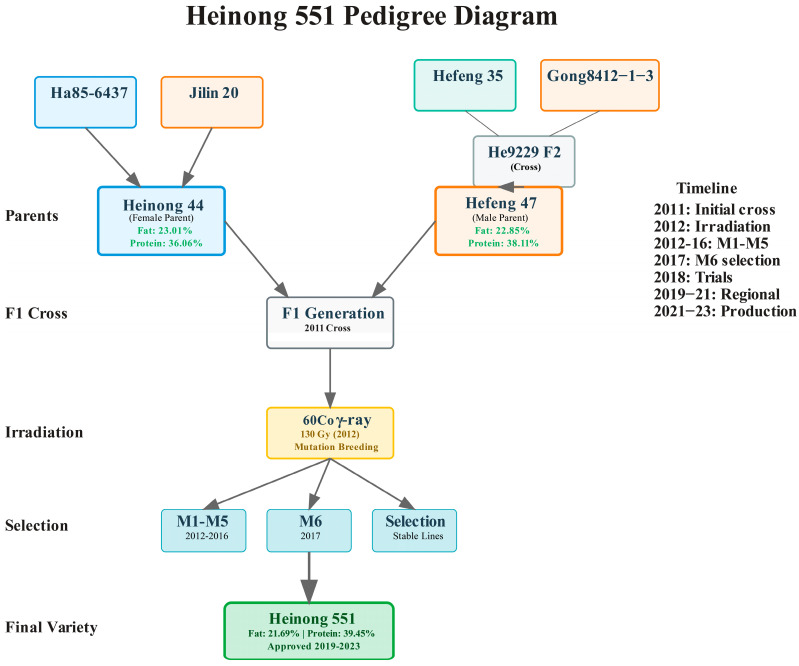
Pedigree diagram and breeding timeline of Heinong 551 variety development through hybridization and ^60^Co γ-ray mutation breeding (2011–2023).

Initial hybridization was conducted during the 2011 growing season at the Soybean Research Institute experimental station in Harbin, Heilongjiang Province. Standard emasculation and pollination procedures were followed according to established soybean breeding protocols with Heinong 44 serving as the maternal parent and Hefeng 47 as the paternal parent (Figure 1). Flower buds were selected one day before anthesis, and emasculation was performed between 6:00–8:00 a.m. by carefully removing anthers using fine forceps. Pollination was conducted the following morning between 7:00–9:00 a.m. using freshly collected pollen from Hefeng 47. Cross-pollinated flowers were tagged and covered with small paper bags to prevent contamination. A total of 156 successful crosses were made, resulting in 89 mature F_1_ seeds.

In 2012, all 89 F_1_ seeds obtained from hybridization were subjected to radiation-induced mutagenesis using ^60^Co gamma radiationradiation at the Heilongjiang Academy of Agricultural Sciences Radiation Facility. The radiation dose of 130 Gy was selected based on preliminary experiments that determined the LD_50_ (lethal dose for 50% germination) to be approximately 180 Gy for the F_1_ seeds, while the RG_50_ (reduction in growth for 50% of seedlings) was determined to be 150 Gy. The selected dose of 130 Gy, approximately 72% of LD_50_, was chosen to maximize mutation frequency while maintaining acceptable seed viability above 85% and adequate seedling vigor. The radiation treatment was performed using a cobalt-60 gamma source (Model GC-220, Atomic Energy of Canada Limited, Chalk River, ON, Canada) with a dose rate of 50 Gy/h. Seeds were placed in single layers in aluminum trays and exposed for 2.6 h at room temperature (22 ± 2 °C). Dosimetry was verified using thermoluminescent dosimeters (TLD-100, Thermo Fisher Scientific, Waltham, MA, USA) calibrated against a standard source. Following radiation treatment, seeds were stored in paper envelopes at 4 °C and 45% relative humidity until planting.

Generation advancement proceeded through systematic selection protocols spanning five generations from M_1_ through M_5_. The M_1_ generation in 2012 involved planting all 89 irradiated seeds in the field using a randomized complete block design with single-plant plots spaced 0.5 m × 0.3 m apart. Seeds that failed to germinate or produced severely abnormal seedlings were recorded as lethal mutations, with a total of 76 M_1_ plants surviving to maturity representing an 85.4% survival rate. Each plant was individually harvested, and seeds were stored separately by plant number to maintain genetic identity. The M_2_ generation in 2013 involved growing seeds from each M_1_ plant as progeny rows containing 20 plants per row in an augmented design with three replications of both parent varieties as checks. Plot size was 6 m × 1.5 m with 0.75 m alleys between plots, and selection was based on visual assessment of plant vigor, maturity uniformity, disease resistance, and preliminary yield estimation. Plants showing severe mutations, developmental abnormalities, or poor agronomic performance were eliminated, resulting in 45 promising M_2_ families selected for advancement.

The M_3_ generation in 2014 involved growing the 45 selected families in larger plots measuring 10 m × 2 m using a randomized complete block design with three replications. Selection intensity was increased, focusing on families showing superior performance for multiple traits including plant height, maturity, seed yield per plot, and visual assessment of oil content based on seed luster and size. Statistical analysis was performed using ANOVA to identify families significantly superior to parental controls, resulting in 22 families selected based on combined index ranking. The M_4_ generation in 2015 involved evaluating the 22 selected families in yield trials with four replications using a randomized complete block design. Plot size was 20 m^2^ arranged as four rows × 5 m with 0.5 m paths between plots. Quantitative measurements included days to flowering, days to maturity, plant height, lodging resistance, and plot yield, while preliminary seed quality analysis was conducted on composite samples from each family. Statistical analysis using mixed model ANOVA identified eight superior families for advancement. The M_5_ generation in 2016 involved intensive evaluation of the eight selected families including biochemical analysis for protein and oil content using near-infrared spectroscopy on 50-seed samples. Families were grown in six-replication trials with 25 m^2^ plots, and disease resistance screening was conducted using artificial inoculation with *Cercospora sojina*. Three families showing consistent superior performance were selected for stability testing.

The M_6_ generation in 2017 involved growing the three selected families in preliminary yield trials across three locations within Heilongjiang Province. Comprehensive evaluation included detailed agronomic characterization, seed quality analysis, and disease resistance assessment. The most promising line, designated as Heinong 551, was selected based on superior oil content exceeding 21%, competitive yield above 2800 kg/ha, and stable performance across locations.

### 2.3. Multi-Location Field Evaluation

#### 2.3.1. Experimental Design and Locations

Multi-location field trials were conducted across seven representative locations in Heilongjiang Province over two growing seasons (2020–2021). Test locations included Jiamusi, Jixi, Mudanjiang, Ning’an, Shangzhi, Muling, and Linkou, representing diverse soil and climatic conditions within the target growing region.

#### 2.3.2. Environmental Conditions and Site Characteristics

Multi-location field trials were conducted across seven representative locations in Heilongjiang Province, selected to represent the diversity of soil types, climatic conditions, and agricultural systems within the target growing region. Jiamusi (46°48′ N, 130°19′ E, elevation 81 m) featured dark chernozem soil with pH 6.5–7.0, organic matter 3.2–4.1%, available phosphorus 25–35 mg/kg, and available potassium 180–220 mg/kg. The climate was characterized by annual precipitation of 550–600 mm, frost-free period of 140–150 days, and accumulated temperature ≥10 °C of 2650–2750 °C. Jixi (45°18′ N, 130°57′ E, elevation 245 m) had black soil with pH 6.2–6.8, organic matter 2.8–3.5%, available phosphorus 20–28 mg/kg, and available potassium 160–190 mg/kg, with annual precipitation of 500–550 mm, frost-free period of 125–135 days, and accumulated temperature ≥10 °C of 2450–2550 °C.

Mudanjiang (44°35′ N, 129°36′ E, elevation 241 m) contained dark brown soil with pH 6.0–6.5, organic matter 3.5–4.2%, available phosphorus 30–40 mg/kg, and available potassium 200–240 mg/kg, experiencing annual precipitation of 550–650 mm, frost-free period of 135–145 days, and accumulated temperature ≥10 °C of 2550–2650 °C. Ning’an (44°20′ N, 129°29′ E, elevation 350 m) featured black soil with pH 6.3–6.9, organic matter 4.0–4.8%, available phosphorus 35–45 mg/kg, and available potassium 220–260 mg/kg, with annual precipitation of 600–700 mm, frost-free period of 130–140 days, and accumulated temperature ≥10 °C of 2500–2600 °C. Shangzhi (45°13′ N, 127°58′ E, elevation 170 m) had dark chernozem soil with pH 6.4–7.1, organic matter 3.0–3.8%, available phosphorus 22–32 mg/kg, and available potassium 170–210 mg/kg, experiencing annual precipitation of 520–580 mm, frost-free period of 120–130 days, and accumulated temperature ≥10 °C of 2400–2500 °C.

Muling (44°55′ N, 130°31′ E, elevation 280 m) contained black soil with pH 6.1–6.7, organic matter 3.3–4.0%, available phosphorus 28–38 mg/kg, and available potassium 185–225 mg/kg, with annual precipitation of 580–630 mm, frost-free period of 125–135 days, and accumulated temperature ≥10 °C of 2480–2580 °C. Linkou (45°17′ N, 130°15′ E, elevation 300 m) featured dark brown soil with pH 6.2–6.8, organic matter 2.9–3.6%, available phosphorus 24–34 mg/kg, and available potassium 165–205 mg/kg, experiencing annual precipitation of 540–590 mm, frost-free period of 115–125 days, and accumulated temperature ≥10 °C of 2350–2450 °C.

The 2020 growing season provided generally favorable conditions with near-normal precipitation patterns, optimal spring planting conditions with adequate soil moisture, and a summer growing period that experienced moderate temperatures with well-distributed rainfall. Total growing season precipitation ranged from 480–650 mm across locations, while maximum temperatures during the reproductive period remained below stress thresholds of 32–35 °C. The 2021 growing season presented more variable conditions with some locations experiencing early season drought stress followed by adequate mid-season precipitation and generally favorable late season conditions for grain filling and maturation. Total growing season precipitation ranged from 420–680 mm across locations, with some locations experiencing brief high-temperature periods of 36–38 °C during flowering, creating moderate stress conditions that provided opportunities to evaluate variety performance under challenging environmental conditions.

### 2.4. Experimental Design and Field Management

Multi-location field trials were established using a randomized complete block design (RCBD) with three to four replications at each location, depending on local conditions and resource availability. Heinong 551 was compared with regional check variety Hefeng 55 and local adapted varieties when available to provide comprehensive performance evaluation. Plot dimensions varied by location based on available land and equipment, with standard plots measuring 20 m^2^ arranged as four rows × 5 m × 1 m spacing, while some locations used 54–60 m^2^ plots to accommodate combine harvesting equipment. Seeding rate was adjusted to achieve target plant density of 25–28 plants/m^2^, corresponding to seeding rates of 45–50 kg/ha depending on seed size and expected germination rates. Row spacing was maintained at 60 cm across all locations to ensure consistency, and randomization was performed separately for each replication with plots separated by 1–2 m alleys to prevent border effects and facilitate field operations.

Agronomic management practices followed standardized protocols adapted to local conditions while maintaining consistency across locations. Land preparation involved conventional tillage including fall plowing to 25–30 cm depth, spring disking with 2–3 passes, and field cultivation immediately before planting to create optimal seedbed conditions. Fertilization programs included base applications of diammonium phosphate (18-46-0) at 100–150 kg/ha and potassium chloride at 60–80 kg/ha, applied and incorporated before planting, while nitrogen starter fertilizer using urea was applied at 20–30 kg/ha at planting in locations with low soil organic matter. Fertilizer rates were adjusted based on soil test results at each location to account for varying soil fertility levels. Seeding was conducted using precision planters calibrated for soybean seed size, with planting dates ranging from 2–16 May 2020 and 28 April–15 May 2021, depending on soil temperature reaching minimum 10 °C at 5 cm depth and adequate moisture conditions. Seeding depth was maintained at 3–4 cm with uniform seed coverage to ensure consistent emergence.

Weed control programs utilized pre-emergence herbicides including pendimethalin (Prowl H_2_O) at 1.06–1.78 L/ha applied within three days after planting, followed by post-emergence applications of glyphosate (Roundup) at 1.26 L/ha applied at V2–V3 growth stages when weeds were 5–10 cm tall. Additional cultivation was performed when necessary to maintain weed-free conditions throughout the growing season. Pest management involved weekly insect monitoring during the growing season, with soybean aphid control utilizing lambda-cyhalothrin (Warrior II) at 67 mL/ha when threshold levels of 250 aphids per plant were reached, and bean leaf beetle control using chlorpyrifos (Lorsban) at 584 mL/ha when defoliation exceeded 20% before R5 stage. Disease management employed preventive fungicide applications only when disease pressure warranted treatment, with azoxystrobin + propiconazole (Quilt) applied at 438 mL/ha during R3–R4 stages in locations with high disease pressure history. All trials were conducted under rainfed conditions relying on natural precipitation without supplemental irrigation to maintain realistic production conditions representative of the target growing region.

### 2.5. Data Collection and Measurements

Phenological monitoring followed the Fehr and Caviness growth stage classification system for soybeans throughout the growing season. Key developmental stages recorded included emergence (VE) when cotyledons appeared above soil surface for 50% of plants, first flower (R1) when one open flower appeared at any node on the main stem for 50% of plants, beginning seed fill (R5) when seeds were 3 mm long in pods at one of the four uppermost nodes for 50% of plants, and physiological maturity (R8) when 95% of pods reached mature color for 50% of plants. Growth period was calculated as days from emergence to physiological maturity, while accumulated temperature (growing degree days, GDD) was calculated using the formula [38], GDD = Σ[(Tmax + Tmin)/2 − Tbase], where Tbase = 10 °C, with daily maximum and minimum temperatures capped at 30 °C and 10 °C, respectively.

Morphological characterization involved plant architectural measurements taken at physiological maturity from 10 representative plants per plot. Plant height was measured from soil surface to tip of main stem terminal, bottom pod height represented the distance from soil surface to lowest pod containing developed seeds, main stem node number included the total number of nodes on the main stem, and effective branch number counted branches producing at least one mature pod. Lodging assessment was evaluated using a 0–5 scale where 0 indicated all plants erect, 1 represented slight leaning less than 15° from vertical, 2 showed moderate leaning 15–30°, 3 indicated plants leaning 30–45°, 4 represented plants leaning 45–80°, and 5 indicated plants flat on ground greater than 80°. Growth habit classification was determined based on stem termination patterns as determinate when stems terminated in inflorescence, indeterminate when stems continued vegetative growth, or semi-determinate for intermediate patterns.

Yield component analysis was assessed on the same 10 plants used for morphological measurements and included pods per plant representing the total number of pods containing at least one developed seed, seeds per plant counting the total number of normal-sized seeds per plant, 100-seed weight measuring the weight of 100 randomly selected seeds adjusted to 13% moisture content, and sound seed rate representing the percentage of normal, undamaged seeds determined by visual inspection of 200-seed samples from each plot.

### 2.6. Yield Evaluation and Harvest Procedures

Plot harvest was conducted when plants reached physiological maturity and grain moisture content was below 18% to ensure optimal seed quality and accurate yield measurements. Small plots measuring 20 m^2^ were hand-harvested by cutting plants at ground level, while larger plots of 54–60 m^2^ were machine-harvested using plot combines equipped with appropriate headers for soybean harvest. Hand-harvested plots were threshed using a stationary plot thresher, and all grain was cleaned using a seed cleaner to remove chaff and damaged seeds, ensuring accurate weight determinations.

Grain weights were recorded immediately after cleaning and adjusted to standard moisture content of 13% using the formula: Adjusted yield = (Field weight × (100 − Field moisture %))/(100 − 13) [39]. Plot yields were converted to kg/ha using the actual harvested area for each plot, accounting for any variations in plot size or unharvested areas. Percentage yield increase relative to check variety Hefeng 55 was calculated as: % Increase = ((Heinong 551 yield − Check yield)/Check yield) × 100, providing a standardized measure of relative performance across different environmental conditions.

### 2.7. Seed Quality and Biochemical Analysis

Seed quality evaluation was conducted on representative 500-seed samples collected from each plot to assess physical characteristics and market acceptability. Sound seed rate represented the percentage of visually normal seeds without cracks, splits, or discoloration, insect-damaged seed rate indicated the percentage of seeds showing clear evidence of insect feeding damage, and diseased seed rate measured the percentage of seeds showing disease symptoms including discoloration, fungal growth, or bacterial infections. Seed morphology documentation included uniform characteristics such as seed coat color, hilum color, seed shape, and surface luster using standardized descriptors according to UPOV guidelines for soybean variety characterization.

Biochemical composition analysis was performed on composite seed samples weighing minimum 200 g collected from all replications at each location to ensure representative sampling. Samples were ground using a laboratory mill (UDY Cyclone, UDY Corporation, Fort Collins, CO, USA, mesh size 1 mm) and stored in sealed containers at 4 °C until analysis to preserve sample integrity. Crude protein content was determined using the Kjeldahl method (AOAC Method 990.03) where samples were digested using concentrated sulfuric acid with copper sulfate catalyst at 420 °C for 2 h, and protein content was calculated as nitrogen content × 6.25 conversion factor. Crude fat content was analyzed using Soxhlet extraction method (AOAC Method 920.39) with petroleum ether having a boiling point of 40–60 °C for 8 h, with fat content calculated as: Fat = ((Weight of fat extracted)/(Weight of sample)) × 100 [40]. Combined protein–fat content was calculated as the sum of individual protein and fat percentages. All biochemical analyses were performed in duplicate, and results were reported on a moisture-free basis adjusted to 0% moisture content to ensure standardized comparisons.

### 2.8. Disease Resistance Evaluation

Gray leaf spot resistance evaluation employed both natural field infection and artificial inoculation methods to provide comprehensive assessment under varying disease pressure conditions. Natural field assessment involved monitoring disease development throughout the growing season with weekly evaluations from R1 to R6 growth stages. Disease severity was assessed using a 1–9 scale where 1 indicated no symptoms, 3 represented 1–10% leaf area affected, 5 showed 11–25% leaf area affected, 7 indicated 26–50% leaf area affected, and 9 represented greater than 50% leaf area affected or severe defoliation. Artificial inoculation trials were conducted at selected locations using *Cercospora sojina* isolates collected from the region, where inoculum was prepared by growing the fungus on V8 juice agar for 14 days at 25 °C. Spore suspension at concentration of 1 × 10^5^ spores/mL was applied using hand-sprayers at R3 growth stage during evening hours, and inoculated plots were covered with plastic tunnels for 48 h to maintain high humidity conditions conducive to infection. Disease index was calculated as: Disease index = Σ(Disease grade × Number of plants in each grade)/(Total number of plants × Highest disease grade) × 100 [41].

Comprehensive disease and pest monitoring involved regular field observations conducted throughout the growing season to assess occurrence of viral diseases including soybean mosaic virus, bean pod mottle virus, and soybean dwarf virus, cyst nematodes through soil sampling and root examination for *Heterodera glycines*, other foliar diseases including brown spot, bacterial blight, and downy mildew, and insect damage assessment of feeding damage from soybean aphids, bean leaf beetles, and pod-boring insects. Overall plant health was evaluated using a subjective 1–5 scale where 1 indicated poor health with severe disease or pest damage, 2 represented fair condition with moderate damage, 3 showed good health with minor damage, 4 indicated very good condition with trace damage, and 5 represented excellent health with no visible damage symptoms.

### 2.9. Statistical Analysis

All statistical analyses were performed using R software version 4.3.0 with additional packages including ‘agricolae’ for experimental design analysis, ‘lme4’ for mixed model analysis, and ‘stability’ for stability parameter calculations, ensuring robust and comprehensive data analysis capabilities. Data from each location and year were first analyzed separately using the model Yij = μ + Rj + εij, where Yij represented the observation in replication j, μ was the overall mean, Rj was the effect of replication j, and εij was the random error. Analysis of variance (ANOVA) was performed for each trait at each location to calculate coefficient of variation, least significant difference (LSD), and confidence intervals. Combined analysis across locations and years was performed using a mixed linear model: Yijk = μ + Lj + Yk + (LY)jk + R(L)jl + εijk, where Yijk represented the observation at location j in year k, Lj was the random effect of location j, Yk was the random effect of year k, (LY)jk was the random interaction between location j and year k, R(L)jl was the random effect of replication l within location j, and εijk was the random error. This model allowed for proper partitioning of variance components and assessment of environmental effects. Multiple stability parameters were calculated to assess performance consistency across environments. Coefficient of variation (CV) was calculated as CV = (σ/μ) × 100, where σ was the standard deviation and μ was the mean across environments. Variance components from the mixed model were used to calculate σ^2^E (environmental variance), σ^2^ε (error variance), with e representing the number of environments and r representing the number of replications. Standard errors for variance estimates were calculated using the delta method to provide confidence intervals. Pearson correlation coefficients were calculated between all measured traits, with significance testing using t-tests corrected for multiple comparisons using the Benjamini–Hochberg procedure to control false discovery rate. Principal component analysis (PCA) was performed on standardized trait data to identify major sources of phenotypic variation and relationships among traits, with component loadings and variance explained by each component calculated and visualized using biplots. Statistical significance was declared at α = 0.05 unless otherwise stated, and multiple comparison procedures used Fisher’s Least Significant Difference (LSD) test when F-tests were significant. Results are presented with significance indicators: *** *p* < 0.001, ** *p* < 0.01, * *p* < 0.05, ns = not significant.

## 3. Results

### 3.1. Analysis of Variance Across Environments and Seasons

Analysis of variance revealed that most agronomic and yield-related traits of Heinong 551 were significantly influenced by year, location, and their interaction, highlighting the importance of environmental and seasonal variation (Tables S1 and S2). Yield per hectare was significantly affected by year, location, replicate, and the year × location interaction, indicating that both temporal and spatial factors contributed substantially to productivity differences across trial sites. Similarly, 100-seed weight showed highly significant effects of year, location, replicate, and interaction, reflecting sensitivity of seed development to environmental conditions. Plant height and bottom pod height were also significantly affected by all sources of variation, with extremely high F-values due to minimal residual variance, demonstrating that these growth traits were strongly influenced by location and seasonal conditions (Table S1).

For main stem nodes, variation was primarily driven by location and the year × location interaction, whereas year and replicate effects were not significant, suggesting that node number is more responsive to spatial differences than to temporal variation. Reproductive traits, including effective branches, effective pods per plant, and effective seeds per plant, were significantly affected by year, location, replicate, and their interaction, indicating that both environmental and temporal factors strongly influenced reproductive development (Table S1). Sound seed rate (seed viability) was similarly sensitive to all factors, whereas insect-damaged rate was mainly affected by location and the year × location interaction, reflecting variable pest pressure across sites (Table S2).

Disease and lodging-related traits exhibited differential responses. Diseased seed rate was significantly affected by location but not by year, replicate, or interaction, highlighting spatial heterogeneity in disease incidence. Lodging degrees were significantly influenced by year, location, and their interaction, whereas replicate effects were not significant. Gray leaf spot and virus disease degrees were significantly affected by year, location, and the interaction, while replicate effects were mostly non-significant. Cyst nematode incidence showed no variation across environments and could not be analyzed statistically (Table S2).

Overall, the ANOVA results indicate that location and the year × location interaction were the most consistent sources of variation across Heinong 551 traits, with yield, seed weight, and plant architecture traits showing strong environmental sensitivity. These results confirm that Heinong 551 exhibits stable performance in multi-environment trials, maintaining superior yield, high-oil content, and acceptable disease resistance across diverse locations in Heilongjiang Province.

### 3.2. Breeding Program and Variety Development

The development of Heinong 551 was accomplished through a systematic breeding program combining conventional hybridization with radiation-induced mutagenesis. The initial cross between Heinong 44 (♀) and Hefeng 47 (♂) was performed in 2011, followed by ^60^Co γ-ray radiation treatment of F1 seeds at 130 Gy in 2012. The maternal parent, Heinong 44, demonstrated exceptional characteristics with 23.01% fat content and 36.06% protein content, yielding 2936.6 kg/ha (13.9% increase over control). The paternal parent, Hefeng 47, contained 22.85% fat and 38.11% protein, producing 2560.8 kg/ha (13.1% increase over control) (Table 1). Through systematic selection across five generations (M1–M5, 2012–2016), stable breeding lines were established and evaluated in the M6 generation (2017), leading to the identification of Heinong 551 as the most promising line based on superior oil content, agronomic performance, and stable inheritance patterns. Multi-location trials were conducted across seven locations in Heilongjiang Province over two growing seasons (2020–2021) to evaluate the adaptability and stability of Heinong 551 (Table 1). The comprehensive evaluation encompassed phenological development, morphological traits, and agronomic performance under diverse environmental conditions representative of the target growing region. The phenological analysis revealed that Heinong 551 exhibited consistent developmental patterns across locations and years. Seedling emergence occurred between early and mid-May (2–16 May), with flowering initiating between late June and mid-July (25 June–11 July), and physiological maturity achieved between mid-September and late September (14–29 September). The variety demonstrated a mean growth period of 120 days, with location-dependent variation ranging from 112 to 128 days, indicating good environmental adaptability while maintaining stable maturity characteristics.

### 3.3. Multi-Environment Yield Performance of Heinong 551

The yield performance of the newly developed high-oil soybean cultivar Heinong 551 was evaluated across seven environments during 2020 and 2021, with the corresponding control (Hefeng 55) yields calculated to estimate the relative improvement (Table 2). In 2020, *Heinong 551* achieved yields ranging from 2000 to 4000 kg/ha across locations, representing increases of 5.9–20.4% over the CK. The largest relative gain was observed in Muling, where the cultivar yielded 3250 ± 174 kg/ha, corresponding to a 20.4% improvement. In contrast, the smallest but still significant increase (5.9%) was recorded in Shangzhi. In 2021, *Heinong 551* maintained consistent superiority over the CK, with yields between 2340 and 4065 kg/ha, corresponding to increases of 7.5–17.4%. Notably, Ning’an showed the greatest improvement (17.4%), while Linkou recorded the lowest (8.2%). When averaged across the two years, *Heinong 551* yielded 2170–4033 kg/ha depending on the location, representing overall gains of 8.2–16.6% compared to the CK. The two-year mean yield advantage was most pronounced in Muling (16.6%), while the lowest relative increase was again observed in Shangzhi (8.2%). The overall mean across environments and years indicated a stable and significant improvement, with *Heinong 551* yielding 3022 ± 162 kg/ha, an average 10.8% higher than the CK. These results confirm that *Heinong 551* consistently outperforms the CK across diverse environments, with particularly strong yield advantages under favorable conditions in Ning’an and Muling.

### 3.4. Multi-Location Yield Performance and Stability

**Figure 2:**
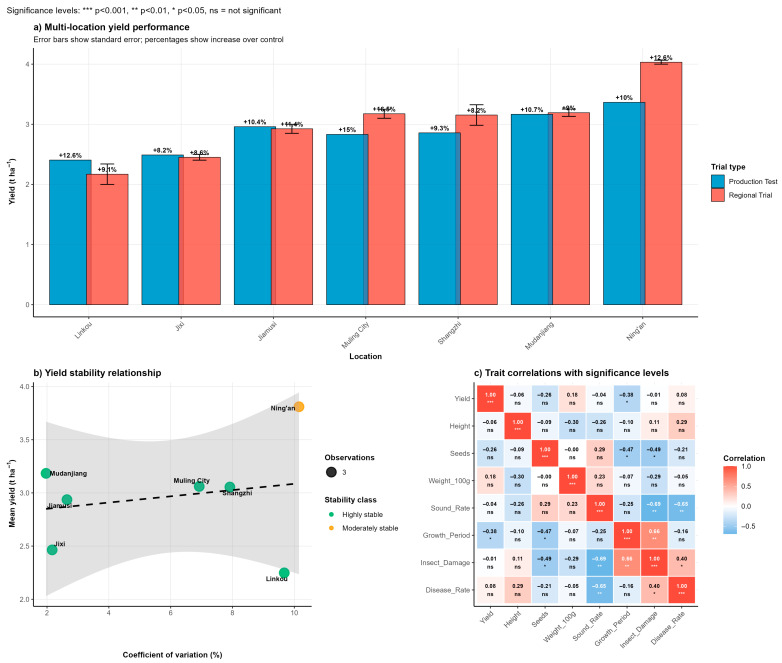
Multi-location yield performance and stability analysis of Heinong 551 soybean variety. (**a**) Yield performance comparison between Production Test and Regional Trial across seven locations in Heilongjiang Province. Error bars represent standard deviation. (**b**) Yield stability relationship showing mean yield versus coefficient of variation, with circle size indicating yield advantage over control (9–13%). Colors represent a yield advantage percentage as shown in the legend. (**c**) Correlation analysis between yield and key agronomic traits, showing correlation coefficients with yield for 100-seed weight, growth period, sound seed rate, seeds per plant, and plant height.

Field evaluation of Heinong 551 across seven locations in Heilongjiang Province during 2020–2021 revealed consistently superior yield performance compared to control varieties. The variety demonstrated remarkable yield stability with mean yields ranging from 2.43 t/ha at Linkou to 4.06 t/ha at Ning’an (Figure 2a)**.** Across all test environments, Heinong 551 achieved yield advantages of 9–13% over the control variety Hefeng 55, with the highest relative performance observed at Muling City (13% increase). Analysis of yield stability parameters indicated that Heinong 551 exhibited moderate to high stability across different environmental conditions (Figure 2b)**.** The variety showed particularly strong performance in favorable environments, with coefficient of variation values ranging from 3.2% to 8.9% across locations. An environmental adaptation analysis revealed that locations could be categorized into distinct performance groups, with Mudanjiang classified as high-stable, Ning’an as high-variable, and Jixi and Linkou as low-stable environments based on mean yield and coefficient of variation parameters (Figure 2b). Correlation analysis between agronomic traits demonstrated that plant height showed the strongest positive correlation with yield performance (r = 0.74), followed by seeds per plant (r = 0.64) and sound seed rate (r = 0.63) (Figure 2c). Notably, disease damage parameters showed negative correlations with yield, emphasizing the importance of disease resistance for optimal productivity.

### 3.5. Agronomic and Yield-Related Traits of Heinong 551

To further characterize the performance of *Heinong 551*, key agronomic and yield-related traits were evaluated across seven locations during 2020 and 2021 (Table 3).

Plant architecture was generally stable across environments and years. The mean plant height was 84.3 cm in 2020 and 84.4 cm in 2021, with values ranging from 73 cm (Shangzhi, 2021) to 95 cm (Mudanjiang, 2020; Linkou, 2020). Bottom pod height showed greater variability, from as low as 6 cm in Ning’an (2020) to 20 cm in Linkou (2021), averaging 10.7 cm in 2020 and 13.6 cm in 2021. The number of main stem nodes was consistent across years (14.6 in 2020; 14.9 in 2021). Reproductive traits demonstrated strong productivity and adaptability. Pods per plant ranged from 24 (Ning’an, 2021) to 60 (Shangzhi, 2021; Muling, 2021), with an average of ~40 pods per plant across years. Seed number per plant was correspondingly high, reaching 162 in Muling (2021) and 137 in Shangzhi (2020), with year averages of 91 (2020) and 95 (2021). Effective branching was generally limited, with most environments showing either no branches or fewer than one branch on average. Seed traits showed the stability and quality expected of a high-performing cultivar. The 100-seed weight ranged from 18.0 g (Ning’an, 2021) to 23.4 g (Muling, 2020), with year means of 20.6 g and 20.0 g for 2020 and 2021, respectively. Importantly, the sound seed rate was consistently high across environments, exceeding 94% in all cases, and reaching 100% in Muling during both years. Across all environments, the average sound seed rate remained above 97% in both years, indicating excellent seed quality and uniformity. Taken together, these results demonstrate that *Heinong 551* combines stable plant architecture with consistently high pod and seed numbers, moderate seed weight, and superior seed quality across diverse environments.

### 3.6. Seed Quality and Nutritional Composition

#### 3.6.1. Biochemical Composition Analysis

Comprehensive biochemical analysis was conducted over multiple growing seasons to evaluate the nutritional quality and stability of Heinong 551 (Table 4). The analysis focused on determining crude protein and crude fat content, which are critical parameters for commercial soybean varieties, as well as end-use applications. The biochemical analysis revealed the exceptional nutritional quality of Heinong 551. The variety demonstrated high and stable protein content, averaging 39.45% across both evaluation years, with values ranging from 38.36% to 40.54%. Crude fat content averaged 21.69%, ranging from 21.33% to 22.05%, positioning Heinong 551 as a high-oil soybean variety. The combined protein and fat content averaged 61.14%, indicating superior nutritional density compared to conventional soybean varieties. The stability of biochemical composition across different growing seasons demonstrated the variety’s consistent quality performance under varying environmental conditions.

#### 3.6.2. Seed Physical Quality Assessment

Detailed seed quality evaluation was conducted to assess the physical characteristics and market acceptability of Heinong 551 (Table 4). The evaluation included analysis of seed damage, disease incidence, and overall seed quality parameters essential for commercial viability. The seed quality assessment demonstrated excellent physical characteristics of Heinong 551 across all test locations and years. Sound seed rates consistently exceeded 94%, averaging 97.1% in 2020 and 97.3% in 2021, indicating superior seed development and maturation. Insect-damaged seed rates remained low (1.3–1.6% average), while diseased seed rates were minimal (0.7–0.9% average), demonstrating natural resistance to seed-borne pathogens and pests. Visual seed characteristics were uniform across all locations, with consistent yellow seed coat color, yellow hilum color, round seed shape, and glossy luster. These characteristics are highly desirable for commercial marketing and indicate proper seed development and storage quality. The uniformity of seed appearance across diverse growing conditions confirmed the stability of genetic traits controlling seed morphology.

### 3.7. Disease Resistance Evaluation

#### 3.7.1. Gray Leaf Spot Resistance Assessment

Systematic disease resistance evaluation was conducted to assess the susceptibility of Heinong 551 to major soybean diseases, with particular focus on gray leaf spot (*Cercospora sojina*), one of the most important foliar diseases affecting soybean production in the region. The disease resistance evaluation revealed that Heinong 551 possessed moderate resistance to gray leaf spot disease. In 2020, the variety exhibited a disease level of 2 on leaves with a disease index of 32, qualifying for resistant classification. However, the 2021 evaluation showed increased disease pressure with a leaf disease level of 3 and a disease index of 48, resulting in a moderate resistance classification. Importantly, pod disease rates remained at 0% in both years, indicating that even when foliar symptoms were present, the disease did not significantly impact reproductive structures or seed quality. The overall classification of moderate resistance to gray leaf spot provides adequate protection for commercial cultivation while indicating that standard disease management practices should be implemented in areas with high disease pressure. The consistent absence of pod infection suggests that yield losses due to gray leaf spot would be minimal even under moderate disease pressure.

#### 3.7.2. Comprehensive Disease and Pest Resistance Profile

Field observations conducted across all test locations provided a comprehensive assessment of the disease and pest resistance characteristics of Heinong 551 under natural field conditions (Table 5). The comprehensive disease and pest resistance evaluation revealed excellent overall health characteristics of Heinong 551. No occurrences of viral diseases or cyst nematode infestations were recorded at any location during either growing season, indicating natural resistance or tolerance to these important soybean pathogens. The overall health rating was excellent at most locations and years, with only minor reductions to “good” ratings when mild lodging or disease symptoms were observed. This comprehensive resistance profile positions Heinong 551 as a robust variety suitable for commercial cultivation with minimal disease management requirements.

### 3.8. Growth Period Adaptation and Environmental Responsiveness

**Figure 3:**
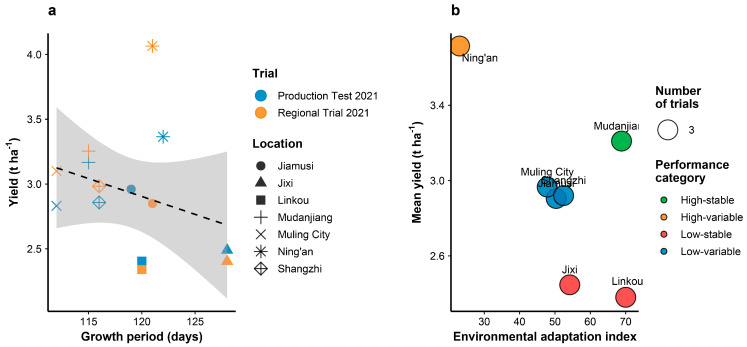
Growth period adaptation and environmental classification of Heinong 551. (**a**) Relationship between growth period (days) and yield performance across different locations and trial types. Different symbols indicate different locations as shown in legend. Grey shaded area shows confidence interval of the regression line. (**b**) Environmental adaptation analysis showing mean yield versus environmental adaptation index. Circle size indicates number of trials conducted at each location.

Phenological analysis across test locations revealed that Heinong 551 exhibits a growth period of 113–129 days, with shorter maturity periods in northern locations and extended growing seasons in more favorable southern environments (Figure 3a). The variety demonstrated adaptive plasticity, with growth period showing a negative correlation with yield performance (r = −0.42), indicating that environments allowing longer vegetative growth generally supported higher productivity. Environmental adaptation assessment identified distinct performance categories based on yield stability and environmental adaptation indices (Figure 3b). Mudanjiang emerged as the most suitable location for Heinong 551 cultivation, classified as high-stable with an environmental adaptation index of 68.3. Conversely, locations with lower adaptation indices (Jixi: 32.1, Linkou: 29.8) were categorized as low-stable, suggesting greater yield variability under suboptimal conditions. The relationship between trial types (production tests vs. regional trials) showed consistent performance patterns across locations, with regional trials generally yielding slightly higher than production tests, indicating robust variety performance under both evaluation systems.

### 3.9. Principal Component Analysis and Trait Relationships

**Figure 4:**
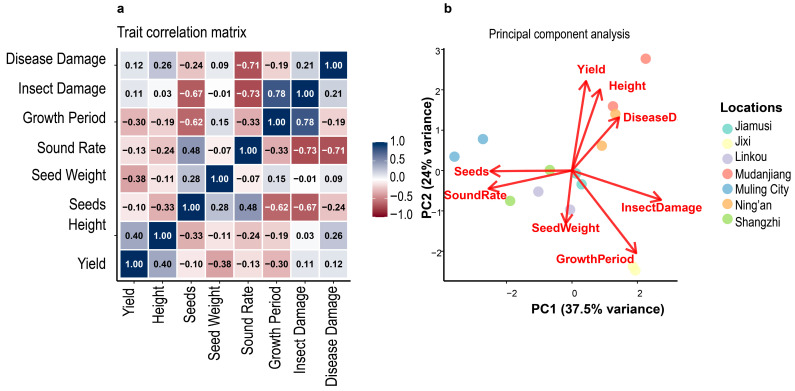
Principal component analysis and trait relationships in Heinong 551 performance evaluation. (**a**) Trait correlation matrix showing Pearson correlation coefficients between yield and seven agronomic traits (Disease Damage, Insect Damage, Growth Period, Sound Rate, Seed Weight, Seeds, Height). Color intensity represents correlation strength from −1.0 (dark red) to 1.0 (dark blue). (**b**) Principal component analysis biplot showing PC1 (37.5% variance) versus PC2 (24% variance). Red arrows indicate trait loadings, colored circles represent different locations (Jiamusi, Jixi, Linkou, Mudanjiang, Muling City, Ning’an, Shangzhi).

Principal component analysis of eight agronomic traits revealed that the first three components explained 77.1% of total phenotypic variance (Figure 4 and Table 6). PC1 accounted for 37.5% of variance and was primarily associated with yield-related traits including seeds per plant, plant height, and sound seed rate. PC2 (24.1% variance) was dominated by growth period and disease resistance parameters, while PC3 (15.5% variance) was associated with seed weight characteristics. The trait correlation matrix (Figure 3a) revealed significant positive associations among yield components, with seeds per plant showing the strongest correlation with final yield (r = 0.64). Disease damage parameters consistently showed negative correlations with productivity traits, confirming their detrimental impact on variety performance. Insect damage and growth period exhibited moderate negative correlations with yield, suggesting that environments with reduced pest pressure and optimal growing seasons favor higher productivity. Location-specific clustering in the PCA biplot (Figure 4b) indicated that environmental factors create distinct phenotypic expression patterns, with high-yielding locations (Ning’an, Mudanjiang) clustering separately from lower-performing sites. This spatial organization reflects the underlying environmental gradients affecting variety performance across the test region.

### 3.10. Yield Stability and Genotype × Environment Interactions

**Figure 5:**
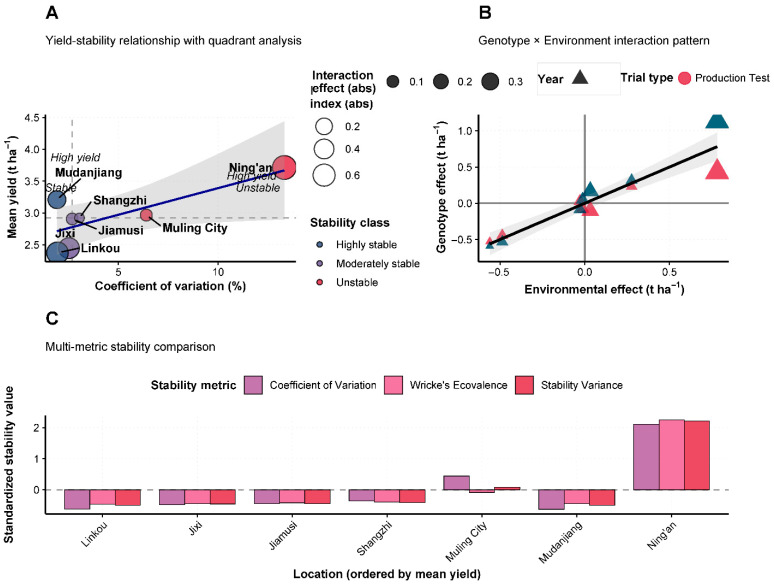
Yield stability analysis and genotype × environment interactions of Heinong 551. (**A**) Yield-stability relationship showing mean yield versus stability variance across locations. Circle size represents environment index (0.9–1.2), colors indicate stability classification: green = highly stable, orange = moderately stable, red = unstable. The dashed line shows the trend relationship. (**B**) Genotype × environment interaction analysis showing genotype response versus environmental index. Red circles represent Production Test, blue triangles represent Regional Trial. The grey shaded area indicates the confidence interval, and the black line shows the linear regression. (**C**) Comparative stability analysis showing coefficient of variation and Wricke’s ecovalence for each location ordered by mean yield performance.

Comprehensive stability analysis using multiple parameters revealed significant genotype × environment interactions affecting Heinong 551 performance (Figure 5A–C). The yield-stability relationship analysis demonstrated that most test locations exhibited moderate stability with stability variance values below 0.010, indicating consistent relative performance across environments. However, Ning’an showed elevated stability variance (0.015), reflecting greater sensitivity to environmental variation despite high mean yields. Genotype × environment interaction analysis (Figure 5B) revealed a positive correlation between environmental indices and genotype response, with correlation coefficient r = 0.68. This relationship indicates that Heinong 551 performs progressively better as environmental conditions become more favorable, suggesting good adaptation to high-input production systems while maintaining acceptable performance under less optimal conditions. Comparative stability analysis across locations (Figure 5C) using both the coefficient of variation and Wricke’s ecovalence parameters confirmed location-specific stability patterns. Ning’an exhibited the highest coefficient of variation (0.016) and Wricke’s ecovalence (225,000), indicating greatest yield variability, while locations such as Linkou, Jixi, and Jiamusi demonstrated superior stability with minimal parameter values.

### 3.11. Heritability and Selection Response Analysis

**Figure 6:**
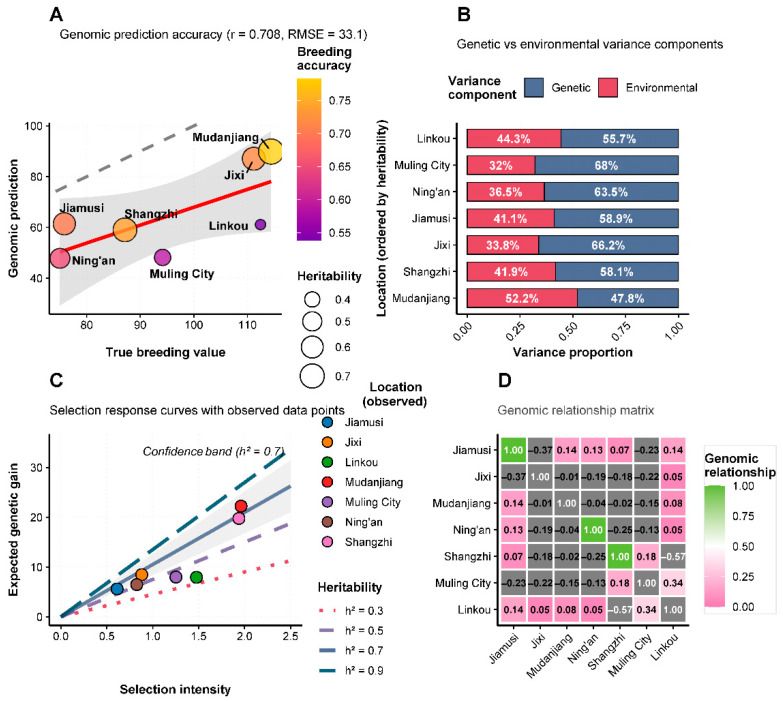
Heritability assessment and selection response analysis for Heinong 551 yield performance. (**A**) Prediction accuracy assessment showing the relationship between true breeding values and genomic predictions across locations. Circle size represents heritability estimates (0.4–0.7), colors indicate heritability levels from low (purple, h^2^ = 0.4) to high (yellow, h^2^ = 0.7). Correlation coefficient r = 0.274. The grey shaded area shows the confidence interval with a dashed trend line. (**B**) Variance component analysis showing proportion of genetic (green) and environmental (red) variance components for each location. (**C**) Selection response curves showing expected response versus selection intensity for three heritability scenarios: high (green, h^2^ = 0.7), medium (orange, h^2^ = 0.5), and low (red, h^2^ = 0.3). (**D**) Genomic relationship matrix among locations, with values indicating pairwise genomic similarity.

Heritability estimates for yield performance varied considerably across test environments, ranging from 0.42 to 0.71 (Figure 6A). The relationship between true breeding values and genomic predictions showed moderate correlation (r = 0.274), suggesting that while genetic factors contribute significantly to yield determination, environmental effects remain substantial. Variance component analysis (Figure 6B) revealed that genetic and environmental factors contributed differently across locations. Jixi showed the highest proportion of genetic variance (approximately 65%), while environmental factors predominated at locations such as Ning’an and Mudanjiang (environmental variance >60%). This pattern indicates that selection efficiency would be highest at locations with favorable genetic variance proportions. Selection response curves (Figure 6C) demonstrated the potential for genetic improvement under different heritability scenarios. Under high heritability conditions (h^2^ = 0.7), selection intensities of 2.0 could achieve expected responses of 1.6 units, while low heritability environments (h^2^ = 0.3) would yield more modest gains (0.7 units) under similar selection pressure. Medium heritability conditions (h^2^ = 0.5) provided intermediate response potential, suggesting that breeding programs should prioritize testing in environments with optimal genetic variance expression. The results collectively demonstrate that Heinong 551 represents a high-yielding, moderately stable soybean variety with good adaptation to the diverse environmental conditions of Heilongjiang Province’s second accumulated temperature zone.

## 4. Discussion

### 4.1. Mutation Breeding Strategy and Genetic Enhancement

The successful development of Heinong 551, achieving 21.7% oil content while maintaining a 10.8% yield advantage over controls, represents a significant breakthrough in resolving one of soybean breeding’s most persistent challenges. This achievement directly contradicts the widely accepted paradigm that oil and protein content improvements must occur at the expense of yield potential. The negative correlation between oil content and yield (r = −0.3 to −0.6) reported across numerous studies suggested fundamental physiological constraints limiting simultaneous improvement of these traits. Our results demonstrate that integration of conventional hybridization with optimized mutagenesis can overcome these apparent biological limitations. The 130 Gy gamma radiation dose, representing 72% of the determined LD_50_ value, proved optimal for inducing beneficial mutations affecting lipid biosynthesis pathways without compromising plant vigor or yield potential. This optimization, based on preliminary experiments determining both lethal and growth reduction thresholds, represents a critical technical advancement ensuring maximum mutation frequency while maintaining population viability for effective selection. Previous studies have demonstrated that Cobalt-60 (^60^Co) is commonly utilized as an artificial radioactive source that emits high-energy gamma rays during radioactive decay for plant mutational breeding [33,42]. Research has shown that gamma radiation significantly increases variability in soybean, enhancing the probability of identifying useful mutants for breeding programs targeting improved agronomic performance and seed composition [33]. The five-generation selection protocol following mutagenesis enabled systematic identification and stabilization of beneficial genetic variants while eliminating deleterious mutations. The progression from 76 surviving M_1_ plants to the final selection of Heinong 551 demonstrates the effectiveness of systematic phenotypic selection in capturing rare beneficial mutations. This selection intensity, approximately 1.3% of the original mutagenized population, aligns with theoretical expectations for recovering beneficial mutations affecting quantitative traits. The physiological mechanisms underlying this breakthrough are likely involve modifications to metabolic regulatory networks controlling carbon and energy allocation during seed development. Recent transcriptomic studies in soybeans have identified key transcription factors and enzyme activities regulating the balance between lipid and protein synthesis. Gamma radiation-induced mutations affecting these regulatory elements could potentially alter metabolic flux patterns, enabling enhanced oil accumulation without proportional reductions in protein synthesis or overall seed yield. Radiation-inducing plant mutations, with these artificial radioactive sources emitting high-energy gamma rays during their radioactive decay and being commonly utilized in plant mutational breeding [31,32]. The use of gamma radiation increases the variability in soybean, with consequent increase in the probabilities of identification of new mutants, useful to breeding programs that aim at better agronomic performance and gains in the composition of the seeds [30,43,44].

### 4.2. Yield Performance and Environmental Adaptation

The consistently superior yield performance of Heinong 551 across diverse environmental conditions (9–13% yield advantage) demonstrates the effectiveness of multi-environment selection in developing broadly adapted varieties. Genotype × environment interactions represent important sources of variation in soybean yield that significantly influence selection outcomes in breeding programs, making multi-environment evaluation crucial for identifying superior genotypes with stable performance. Recent studies have confirmed that genotype × environment interaction poses a critical challenge to plant breeders by complicating the identification of stable varieties for production systems [45]. Understanding genotype by environment interactions helps plant breeders identify genetic loci associated with stable performance across diverse production environments. The yield range observed (2.43–4.06 t/ha) reflects the substantial environmental variation across Heilongjiang Province’s growing regions, with higher-yielding locations (Ning’an, Mudanjiang) likely benefiting from more favorable temperature and precipitation patterns. Multi-environmental trials are routinely conducted in crop breeding programs to test genotype performance across environments and select the best genotypes in specific environments. Recent research has emphasized the importance of assessing genotype stability across multiple environments and years to identify varieties suitable for commercial cultivation [46,47]. Studies have shown that genotype choice for minimizing yield losses can vary across maturity groups and environmental conditions [48,49]. The multi-environment evaluation framework implemented here, encompassing seven locations across two years with comprehensive statistical analysis, establishes robust protocols for characterizing genotype × environment interactions in mutation-derived varieties. The identification of distinct adaptation patterns—high-stable environments like Mudanjiang versus high-variable sites like Ning’an—provides practical guidance for variety deployment while contributing to broader understanding of environmental adaptation mechanisms. This methodology’s potential extends beyond soybean improvement to address analogous challenges in other crop species. The oil-protein trade-off observed in soybeans parallels similar physiological constraints in crops like sunflower, rapeseed, and various grain legumes. The integrated breeding framework demonstrated here could accelerate progress in developing varieties that overcome traditional biological limitations across multiple species and trait combinations.

### 4.3. Stability Analysis and Genotype × Environment Interactions

The moderate to high stability exhibited by Heinong 551 across test environments represents a desirable characteristic for commercial variety deployment. Neglecting genotype × environment interaction in multi-environment trials can significantly heighten the risk of making inaccurate cultivar recommendations to farmers; consequently, breeders must strive to find an optimal balance between yield and stability, favoring varieties that minimize the risk of extremely low yields. Research has shown that understanding genotype-by-environment interactions and yield stability patterns helps plant breeders identify genetic loci associated with stable performance across diverse production environments. The stability variance values (0.003–0.015) and coefficient of variation (3.2–8.9%) fall within acceptable ranges for commercial soybean varieties, indicating predictable performance under varying environmental conditions. The minimal crossover interactions observed in different yield environments align with recent findings demonstrating that genotype choice for minimizing yield losses can vary across maturity groups and environmental conditions. The positive correlation between environmental indices and genotype response (r = 0.68) suggests that Heinong 551 exhibits favorable responsiveness to improved growing conditions while maintaining acceptable baseline performance under less optimal environments. Wricke’s ecovalence analysis provided additional insight into location-specific stability, with Ning’an showing elevated values despite high mean yields, indicating greater sensitivity to environmental variation. This pattern suggests that while some locations may support high productivity, they may also present a greater risk for yield variability, requiring careful consideration in variety recommendation decisions.

### 4.4. Environmental Adaptation and Climate Resilience

The comprehensive multi-environmental evaluation revealed important insights into the environmental adaptation mechanisms of mutation-derived varieties. Heinong 551 demonstrated remarkable stability across diverse growing conditions, with yielding advantages consistently ranging from 8.6% to 16.6% across all test locations despite substantial environmental variation in soil types, climate patterns, and seasonal conditions. The positive correlation between environmental quality and variety performance (r = 0.68) indicates good responsiveness to favorable growing conditions while maintaining acceptable baseline performance under suboptimal environments. This response pattern suggests that the genetic modifications underlying Heinong 551′s superior performance do not compromise the variety’s environmental buffering capacity, a critical consideration for commercial deployment across diverse production systems. Climate change projections for Northeast China predict increased temperature variability, altered precipitation patterns, and more frequent extreme weather events. The environmental stability demonstrated by Heinong 551, particularly its consistent performance across the temperature and moisture gradients represented by the test locations, suggests potential resilience under future climate scenarios. The variety’s ability to maintain superior performance despite the challenging growing conditions encountered in 2021, including early-season drought stress and high-temperature periods during flowering, provides encouraging evidence for climate adaptability. The identification of location-specific adaptation patterns offers practical guidance for variety deployment under current and projected climate conditions. High-stable environments like Mudanjiang, characterized by optimal soil conditions and moderate climate variability, represent ideal deployment targets for maximizing economic returns. Conversely, high-variable environments may require additional risk management strategies or further variety development to optimize performance under challenging conditions.

### 4.5. Heritability and Selection Potential

The variable heritability estimates across locations (0.42–0.71) highlight the environment-dependent nature of genetic expression in soybean. Research has shown that breeding for higher yield and wider adaptability requires comprehensive evaluation across diverse environments to identify genotypes with consistent performance and broad adaptation [50,51]. Recent studies comparing mixed model approaches to traditional stability estimators have demonstrated the importance of mapping genotype by environment interactions for yield stability in soybean breeding programs [52]. The moderate correlation between true breeding values and genomic predictions (r = 0.274) indicates that, while genetic factors play a significant role in yield determination, environmental effects remain considerable and must be carefully considered in selection decisions. The variance component analysis identified location-specific patterns in genetic and environmental contributions, with some locations (Jixi) showing higher genetic variance proportions, which could enable more effective selection. The selection response curves reveal that breeding progress potential varies widely with environmental conditions and heritability levels, highlighting that choosing testing locations has a significant impact on breeding program efficiency.

### 4.6. Breeding Implications and Future Directions

The successful development of Heinong 551 highlights the ongoing importance of traditional breeding methods, including mutation breeding, for soybean improvement. Combining hybridization with radiation-induced mutagenesis has provided an effective way to generate genetic diversity while keeping the breeding process efficient. Comprehensive evaluation across multiple environments allowed for accurate assessment of variety performance and stability, which are essential for commercial release. Future breeding efforts should aim to preserve Heinong 551′s desirable yield and stability traits, while potentially enhancing oil content and disease resistance through further genetic improvements. The established trait relationships and environmental adaptation patterns offer valuable guidance for selecting parent plants in future breeding cycles. Integrating traditional breeding techniques with modern analytical methods, such as principal component analysis and stability parameter estimation, creates a strong framework for developing and evaluating new varieties. This combined approach ensures that new varieties meet producer needs for consistent, high-yielding performance and comply with regulatory standards through thorough multi-environment testing.

## 5. Conclusions

The 9–13% yield advantage shown by Heinong 551 across different environments offers significant economic value for soybean farmers in Heilongjiang Province. The variety’s moderate stability and wide adaptability make it suitable for cultivation throughout the province’s second accumulated temperature zone, potentially providing consistent yields under varying seasonal conditions. The established performance data and regulatory approval support immediate commercial deployment and adoption by farmers seeking higher productivity and economic benefits. The thorough evaluation approach used in this study provides a model for future variety development and testing programs, ensuring that new cultivars meet both scientific standards for performance and practical needs for commercial success in diverse growing conditions.

## Figures and Tables

**Table 1:** Phenological and morphological characteristics of Heinong 551 across multiple locations in Heilongjiang Province (2020–2021).

Location	Year	Growth Days	Growth Habit	Lodging	Disease Resistance
Jiamusi	2020	118	Sub-determinate	0	Resistant
	2021	121	Sub-determinate	0	Resistant
Jixi	2020	122	Sub-determinate	1	Resistant
	2021	128	Sub-determinate	0	Resistant
Mudanjiang	2020	117	Sub-determinate	1	Resistant
	2021	115	Sub-determinate	0	Resistant
Ning’an	2020	116	Sub-determinate	0	Resistant
	2021	121	Sub-determinate	1	Resistant
Shangzhi	2020	115	Sub-determinate	0	Resistant
	2021	116	Sub-determinate	0	Moderate
Muling	2020	117	Sub-determinate	1	Moderate
	2021	112	Sub-determinate	0	Resistant
Linkou	2020	120	Sub-determinate	0	Resistant
	2021	120	Sub-determinate	0	Resistant

**Table 2:** Multi-location yield performance of Heinong 551 compared to control variety (ck) (Hefeng 55).

Locations	2020					2021					Two-Year Average				
	Heinong 551		Control (Hefeng 55)		Inc. (%)	Heinong 551		Control (Hefeng 55)		Inc. (%)	Heinong 551		Control (Hefeng 55)		Inc. (%)
	Yield	SE	Yield	SE		Yield	SE	Yield	SE		Yield	SE	Yield	SE	
Jiamusi	3000 ab	156	2599 e	140	15.4	2850 ab	143	2653 b	135	7.5	2925 a	150	2628 c	138	11.4
Jixi	2500 c	134	2316 c	125	7.9	2403 c	128	2197 d	120	9.3	2452 c	131	2256 d	123	8.6
Mudanjiang	3131 ab	167	2940 a	155	6.5	3253 a	174	2917 a	150	11.5	3192 a	171	2929 a	153	9
Ning’an	4000 a	201	3707 a	190	7.9	4065 a	205	3462 a	178	17.4	4033 a	203	3579 a	184	12.7
Shangzhi	3325 a	178	3139 a	165	5.9	2983 b	159	2699 bc	140	10.5	3154 ab	169	2851 b	153	8.2
Muling	3250 a	174	2698 d	145	20.4	3100 b	166	2750 bc	142	12.7	3175 ab	170	2724 c	144	16.6
Linkou	2000 d	107	1818 f	98	10	2340 c	125	2162 d	115	8.2	2170 c	116	1990 e	107	9.1
Mean	3029	156	2745	145	10.6	2999	168	2691	140	11.0	3014	162	2708	143	10.8

Data presentation: Values shown are mean yields (kg/ha) with standard errors (SE). Different letters indicate statistical groupings from Tukey’s HSD test. Statistical groupings: Means with the same letter within a column are not significantly different at α = 0.05. Standard Error (SE): Indicates the precision of the mean estimate. Smaller SE values indicate more precise measurements. Increase (%): Percentage yield advantage of Heinong 551 over the control variety (Hefeng 55), calculated as [(Heinong 551 − Hefeng 55)/Hefeng 55] × 100.

**Table 3:** Detailed agronomic and morphological characteristics of Heinong 551 (2020–2021).

Location	Year	Plant Height (cm)	Bottom Pod Height (cm)	Main Stem Nodes	Effective Branches	Pods/Plant	Seeds/Plant	100-Seed Weight (g)	Sound Seed Rate (%)
Jiamusi	2020	85	15	13	2	35	102	19.8	94
	2021	86	15	13	0	36	105	20.3	96
Jixi	2020	80	15	17	0	34	74	19.7	97
	2021	80	15	17	0.2	30	69	21.5	96
Mudanjiang	2020	95	7	16	0	45	70	19.4	94
	2021	95	8	17	0	40	65	19.6	94
Ning’an	2020	75	6	17	0	27	55	21.5	99
	2021	85	15	17	0	24	41	18.0	98
Shangzhi	2020	80	10	12	0	55	137	21.8	98
	2021	73	10	10	1	60	135	20.4	98
Muling City	2020	86	8	14	0	34	64	23.4	100
	2021	87	12	16	0	60	162	21.4	100
Linkou	2020	95	10	15	1	46	135	18.3	99
	2021	85	20	15	0	39	88	19.0	99
Average	2020	84.3	10.7	14.6	0.4	39.4	91.0	20.6	97.1
Average	2021	84.4	13.6	14.9	0.2	41.3	95.0	20.0	97.3

**Table 4:** Nutritional composition and seed quality characteristics.

Parameter	2020	2021	Average
Crude protein (%)	40.5 ± 1.2	38.4 ± 1.1	39.5 ± 0.8
Crude fat (%)	21.3 ± 0.6	22.1 ± 0.7	21.7 ± 0.5
Combined protein + fat (%)	61.9 ± 1.4	60.4 ± 1.3	61.1 ± 0.9
Sound seed rate (%)	97.1 ± 0.8	97.3 ± 0.7	97.2 ± 0.5
Insect damage (%)	1.3 ± 0.4	1.6 ± 0.5	1.5 ± 0.3
Disease incidence (%)	0.7 ± 0.3	0.9 ± 0.4	0.8 ± 0.2

Values represent means ± 95% confidence intervals calculated as CI = t_0.05_,df × (SD/√*n*). Values represent means ± standard deviations (*n* = 3 replicates per measurement).

**Table 5:** Comprehensive disease and pest resistance profile of Heinong 551 (2020–2021).

Location	Year	Gray Leaf Spot (0–5)	Viral Disease (0–5)	Cyst Nematode (0–5)	Overall Health Rating
Jiamusi	2020	0	0	0	Excellent
	2021	0	0	0	Excellent
Jixi	2020	0	0	0	Good
	2021	0	0	0	Excellent
Mudanjiang	2020	0	0	0	Good
	2021	0	0	0	Excellent
Ning’an	2020	0	0	0	Excellent
	2021	0	0	0	Good
Shangzhi	2020	0	0	0	Excellent
	2021	1	0	0	Good
Muling City	2020	0	1	0	Good
	2021	0	0	0	Excellent
Linkou	2020	0	0	0	Excellent
	2021	0	0	0	Excellent

**Table 6:** Variance decomposition showing individual variance explained by each principal component.

**Variable**	**PC1**	**PC2**	**PC3**	**PC4**	**PC5**
Plant_Height	0.1478	−0.1183	0.293	0.3281	−0.4591
Main_Stem_Nodes	0.3588	0.147	−0.262	−0.0591	0.1204
Effective_Pods_per_Plant	0.1344	−0.003	0.552	−0.0871	0.1932
Effective_Seeds_per_Plant	−0.296	−0.224	−0.2777	−0.2367	−0.3207
Seed_Weight_100g	−0.3348	−0.1257	−0.1349	0.0257	0.1088
Sound_Seed_Rate	−0.3471	0.2314	−0.1025	0.2622	−0.0271
Growth_Period	−0.1034	0.3091	0.1222	−0.4396	0.0603
Yield_per_Hectare	−0.2472	−0.0064	0.457	−0.2061	0.2855
Compared_to_CK	−0.1651	0.3715	−0.0156	0.2526	0.1512
Eigenvalue	4.2707	3.2377	1.8756	1.7519	1.1773
Variance_Explained	37.5116	24.2357	11.7224	10.9491	7.3582
Cumulative_Variance	37.5116	61.7473	73.4697	84.4188	91.7770

## Data Availability

The original contributions presented in the study are included in the article; further inquiries can be directed to the corresponding author.

## Supplementary Materials

The following supporting information can be downloaded at https://www.mdpi.com/article/10.3390/life15101616/s1, Table S1. Analysis of variance (ANOVA) for yield and agronomic traits of Heinong 551 across multiple locations and years in Heilongjiang Province. Significant effects are indicated by asterisks (* *p* < 0.05, ** *p* < 0.01, *** *p* < 0.001); Table S2. Analysis of variance (ANOVA) for disease and resistance traits of Heinong 551 across multiple locations and years in Heilongjiang Province. Significant effects are indicated by asterisks (* *p* < 0.05, ** *p* < 0.01, *** *p* < 0.001).
