# Supplementary material for: Improvement of Premium Oil Soybean Variety Heinong 551 with Integrating Conventional Hybridization and Gamma Radiation
Source: Life (Basel). 2025 Oct 16;15(10):1616. doi: 10.3390/life15101616 (PMC12565423; doi:10.3390/life15101616)
Supplement: Supplementary file 1 [file life-15-01616-s001.zip › life-3905900-supplementary.pdf]

Table S1. Analysis of variance (ANOVA) for yield and agronomic traits of Heinong 551 across multiple locations and years in Heilongjiang Province. Significant effects are indicated by asterisks (*p < 0.05, **p < 0.01, ***p < 0.001).

Trait	Source	DF	SS	MS	F-value	P-value	Variance Explained (%)	Significance
Yield per Hectare	Year	1	27.26	27.26	3.59	0.065	0.16	NS
	Location	6	1,174.30	195.72	25.76	<0.001	6.75	***
	Replicate	3	2,506.93	835.64	109.99	<0.001	14.41	***
	Year × Location	6	802.67	133.78	17.61	<0.001	4.61	***
	Residual	39	296.24	7.60	-	-	1.70	-
**Compared to CK (%)**	Year	1	0.29	0.29	0.14	0.713	0.02	NS
	Location	6	1,226.18	204.36	96.17	<0.001	85.53	***
	Replicate	3	5.89	1.96	0.92	0.439	0.41	NS
	Year × Location	6	117.57	19.59	9.22	<0.001	8.20	***
	Residual	39	82.86	2.12	-	-	5.78	-
100-Seed Weight (g)	Year	1	4.80	4.80	4,848.48	<0.001	4.86	***
	Location	6	34.83	5.81	5,858.59	<0.001	35.27	***
	Replicate	3	1.98	0.66	666.67	<0.001	2.00	***
	Year × Location	6	57.14	9.52	9,616.16	<0.001	57.85	***
	Residual	39	0.039	0.001	-	-	0.04	-
Plant Height (cm)	Year	1	28.57	28.57	18.92	<0.001	1.37	***
	Location	6	1,169.71	194.95	129.08	<0.001	56.13	***
	Replicate	3	84.00	28.00	18.54	<0.001	4.03	***
	Year × Location	6	807.43	134.57	89.12	<0.001	38.75	***
	Residual	39	58.89	1.51	-	-	2.83	-
Bottom Pod Height (cm)	Year	1	64.29	64.29	47.62	<0.001	9.86	***
	Location	6	427.43	71.24	52.77	<0.001	65.56	***
	Replicate	3	28.00	9.33	6.91	<0.001	4.30	***
	Year × Location	6	125.71	20.95	15.52	<0.001	19.28	***
	Residual	39	5.26	0.13	-	-	0.81	-
Main Stem Nodes	Year	1	1.60	1.60	0.69	0.412	0.48	NS
	Location	6	262.86	43.81	18.88	<0.001	79.10	***
	Replicate	3	3.01	1.00	0.43	0.731	0.91	NS
	Year × Location	6	56.00	9.33	4.02	0.003	16.85	**
	Residual	39	9.06	0.23	-	-	2.73	-
Effective Branches	Year	1	0.011	0.011	1.90	0.176	0.04	1
	Location	6	9.10	1.52	262.07	<0.001	31.08	6
	Replicate	3	0.116	0.039	6.72	<0.001	0.40	3
	Year × Location	6	20.07	3.35	577.59	<0.001	68.52	6
	Residual	39	0.226	0.006	-	-	0.77	39
Effective Pods per Plant	Year	1	34.57	34.57	205.77	<0.001	0.59	***
	Location	6	4,439.43	739.91	4,403.98	<0.001	75.50	***
	Replicate	3	33.43	11.14	66.31	<0.001	0.57	***
	Year × Location	6	1,311.43	218.57	1,300.90	<0.001	22.30	***
	Residual	39	6.55	0.17	-	-	0.11	-
Effective Seeds per Plant	Year	1	28.57	28.57	104.62	<0.001	0.08	***
	Location	6	25,379.18	4,229.86	15,492.53	<0.001	67.17	***

Replicate	3	167.86	55.95	204.95	<0.001	0.44	***
Year × Location	6	12,271.18	2,045.20	7,492.67	<0.001	32.47	***
Residual	39	10.65	0.27	-	-	0.03	-

Table S2. Analysis of variance (ANOVA) for disease and resistance traits of Heinong 551 across multiple locations and years in Heilongjiang Province. Significant effects are indicated by asterisks (*p < 0.05, **p < 0.01, ***p < 0.001).

Trait	Source	DF	SS	MS	F-value	P-value	Variance Explained (%)	Significance
Sound Seed Rate (seed viability) (%)	Year	1	0.29	0.29	0.13	0.722	0.08	NS
	Location	6	128.18	21.36	9.36	<0.001	35.34	***
	Replicate	3	13.89	4.63	2.03	0.125	3.83	NS
	Year × Location	6	129.57	21.59	9.46	<0.001	35.72	***
	Residual	39	88.96	2.28	-	-	24.53	-
Insect Damaged Rate (%)	Year	1	0.29	0.29	28.57	<0.001	2.94	***
	Location	6	6.18	1.03	102.86	<0.001	62.75	***
	Replicate	3	0.54	0.18	17.86	<0.001	5.45	***
	Year × Location	6	2.46	0.41	41.00	<0.001	24.97	***
	Residual	39	0.39	0.010	-	-	3.96	-
Diseased Seed Rate (%)	Year	1	0.29	0.29	2.64	0.112	1.47	NS
	Location	6	18.18	3.03	27.55	<0.001	92.31	***
	Replicate	3	0.54	0.18	1.64	0.195	2.74	NS
	Year × Location	6	0.46	0.076	0.69	0.658	2.33	NS
	Residual	39	4.29	0.11	-	-	21.78	-
**Growth Period (days)**	Year	1	12.11	12.11	54.86	<0.001	3.99	***
	Location	6	257.71	42.95	194.55	<0.001	84.95	***
	Replicate	3	2.96	0.99	4.48	0.008	0.98	**
	Year × Location	6	22.18	3.70	16.75	<0.001	7.31	***
	Residual	39	8.61	0.22	-	-	2.84	-
**Emergence (days)**	Year	1	0.046	0.046	0.83	0.367	0.18	NS
	Location	6	19.43	3.24	58.45	<0.001	75.83	***
	Replicate	3	0.19	0.063	1.14	0.343	0.74	NS
	Year × Location	6	3.79	0.63	11.39	<0.001	14.79	***

	Residual	39	2.16	0.055	-	-	8.43	-
**Flowering (days)**	Year	1	0.23	0.23	1.59	0.215	0.19	NS
	Location	6	112.64	18.77	130.07	<0.001	95.27	***
	Replicate	3	0.54	0.18	1.25	0.304	0.46	NS
	Year × Location	6	0.93	0.15	1.07	0.395	0.79	NS
	Residual	39	5.63	0.14	-	-	4.76	-
**Maturity (days)**	Year	1	0.0011	0.0011	0.052	0.820	0.08	NS
	Location	6	0.098	0.016	0.77	0.597	7.27	NS
	Replicate	3	0.0089	0.0030	0.14	0.936	0.66	NS
	Year × Location	6	0.098	0.016	0.77	0.597	7.27	NS
	Residual	39	0.83	0.021	-	-	61.59	-
DISEASE RESISTANCE	All observations = 0	-	0	0	NA	NA	NA	NS

Significant effects are indicated by asterisks (*p < 0.05, **p < 0.01, *p < 0.001).NA .nun significant.
